# Computer-Guided Biopsy of Osteosclerotic Jaw Lesion Using 3D-Printed Surgical Guides: A Fully Digital Workflow

**DOI:** 10.1097/SCS.0000000000012501

**Published:** 2026-01-30

**Authors:** Pierluigi Mariani, Diana Russo, Francesco Rullo, Lucio Lo Russo, Angelo Salamini, Vincenzo Ronsivalle, Marco Cicciù, Luigi Laino

**Affiliations:** * Multidisciplinary Department of Medical-Surgical and Odontostomatological Specialties, University of Campania, “Luigi Vanvitelli”, Naples; † Department of Clinical and Experimental Medicine, School of Dentistry, University of Foggia, Foggia; ‡ R&D Manager Evolab, Caserta; § Department of General Surgery and Surgical-Medical Specialties, School of Dentistry, University of Catania, Catania, Italy

**Keywords:** 3D-printed surgical guide, AI-based CBCT segmentation, computer-guided biopsy, fully digital workflow, osteosclerotic jaw lesions

## Abstract

Osteosclerotic jaw lesions, often incidentally detected on routine radiographs, are rarely biopsied due to their benign appearance and proximity to delicate anatomic structures. This case report presents a fully digital workflow for guided biopsy of a deep mandibular osteosclerotic lesion, integrating artificial intelligence-based segmentation, intraoral scanning, CAD design, and 3D printing of surgical templates. A 3D-printed guide with depth-control stops was used to safely and precisely position trephine burs between the roots of tooth 4.6 and the mandibular canal. The approach allowed accurate tissue sampling for histopathologic diagnosis (osteoma), minimizing invasiveness and risk to adjacent structures. Postoperative CBCT confirmed the accuracy of the biopsy, with deviations of 0.6 mm (linear), 4 degrees (angular), and −0.2 mm (depth). This technique demonstrates the potential of artificial intellingence-assisted digital planning and 3D printing to enhance biopsy precision for intraosseous lesions.

Asymptomatic osteosclerotic lesions, often incidentally detected during routine radiographic examinations, are rarely investigated further due to their usually benign nature, particularly when they are near critical anatomic structures. This approach may result in these lesions remaining without a definitive diagnosis or being classified solely based on a presumptive radiographic interpretation.

Performing an incisional biopsy on such lesions can be challenging, both due to their location and the complex anatomy of the maxillofacial region, which is characterized by delicate structures such as the inferior alveolar nerve and dental roots.^1^ In this context, 3-dimensional (3D) printed surgical guides may represent valuable tools to enhance biopsy accuracy and ensure the precise collection of tissue samples for histological analysis.^2–4^

The 3D printing is already widely used in oral and maxillofacial surgery, particularly in implantology, where the use of surgical guides has become standard practice.^5^ However, with regard to jawbone pathologies, the current literature remains limited and fragmented. A recent systematic review,^6^ aimed at evaluating the contribution of CAD/CAM technology to the surgical management of jaw cysts and tumors, included only 13 studies, of which only one was a randomized clinical trial (RCT). The findings revealed that 3D printing has mainly been utilized for the fabrication of anatomic models for preoperative planning and visualization, as well as for educational purposes. Only 8 studies reported the intraoperative use of surgical guides, mainly for intraoperative osteotomy and, in fewer cases, for intraoperative drilling location, whereas a single study described the application of 3D-printed postoperative devices for the marsupialization of osteolytic lesions.^7^ More recent studies have provided preliminary data on the accuracy of guided biopsies compared with freehand approaches, highlighting the superior precision of the former.^8,9^ Nonetheless, to date, the literature does not yet provide a detailed, standardized, and reproducible protocol for fully digital surgical planning. The aim of this paper is to clearly and in detail report the full digital workflow for performing computer-guided biopsies of osteosclerotic lesions.

## MATERIALS AND METHODS

**FIGURE 1:**
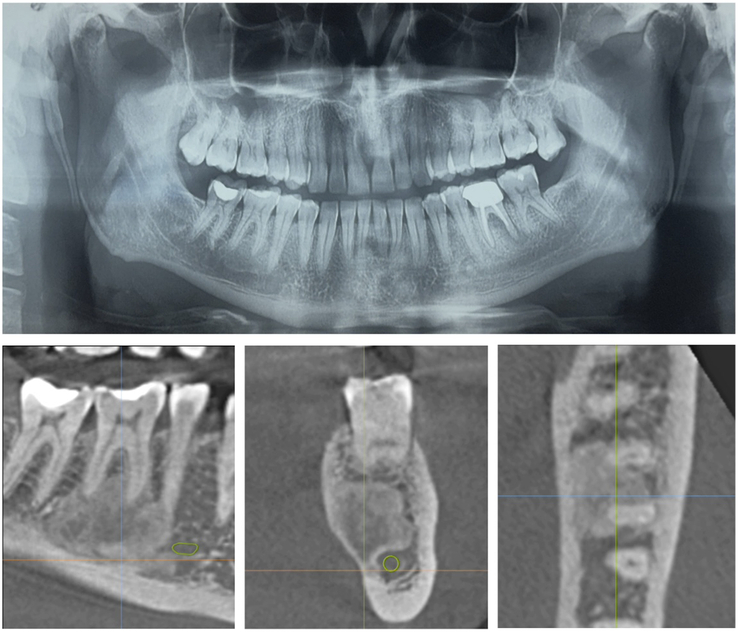
Radiographic images: orthopantomography, and coronal, axial, and panorex CBCT sections of the osteosclerotic lesion.

A 38-year-old man was referred by the dentist to the Department of Oral Surgery of the University of Campania Luigi Vanvitelli to investigate the nature of an osteosclerotic lesion, localized at the level of teeth 4.5 and 4.6, having relationships with the apices of these teeth and apically had relationships with the mandibular canal (MC) (Fig. 1).

The area chosen for biopsy was between the roots of tooth 4.6 and coronal to the MC. The use of 2 coaxial trephine burs was planned, one 5 mm in external diameter and the other 4 mm. (Meisinger T229L-080-RAL and 229L-030-RAL; Hager & Meisinger GmbH).

**FIGURE 2:**
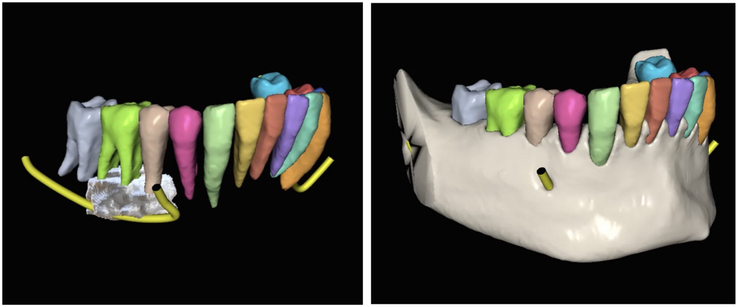
The automatic segmentation function using artificial intelligence.

Using Blue Sky Plan software [Blue Sky Bio, LLC. (2025). *Blue Sky Plan* (Dental treatment planning software)],^10^ the intraoral scan (STL) file was aligned with the DICOM data from the Cone Beam computed tomography analysis [Orthopos SL (Dentsply Sirona)]. The automatic segmentation function using Artificial Intelligence (AI) was used to segment the mandibular bone, the teeth, and the MC (Fig. 2). The osteosclerotic lesion to be analyzed was manually segmented by applying a filter related to the range between a maximum and minimum gray value according to the Hounsfield scale.

An STL file of each component was then obtained, useful for the case study. Obtaining individual files of the different components was useful for surgical planning as it allowed for selective visualization of each component. The chosen trephine burs were designed in 3D using Rhinoceros CAD [Robert McNeel & Associates. *Rhinoceros* (software): Robert McNeel & Associates; 2025].

**FIGURE 3:**
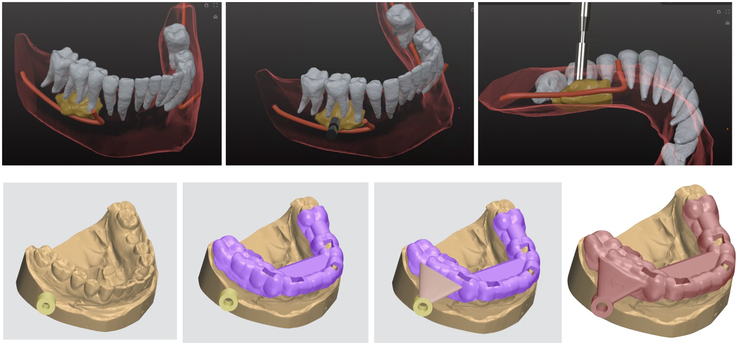
Digital planning using the CAD design of the bur imported along with all the anatomic components into Nomad Sculpt software. Design of the surgical guide and bur sleeve using 3Shape Appliance Designer software.

All files were loaded into Nomad Sculpt software [Ginier S. *Nomad Sculpt* (software): Stéphane Ginier; 2025]. Nomad Sculpt was used for its advanced visualization functions and for better management of movements and rotations between files. The direction and depth of the trephine burs entry were established, also considering the use of an extension (Fig. 3). Once the final position of the burs was validated, all files were exported in position and loaded into 3Shape Appliance Designer software [3Shape A/S. *3Shape Appliance Designer* (software); 2025] (Fig. 3). This kind of support can also be printed by the Form4BL 3D printer (Formlabs Inc.)

The portion of the template relating to the dental support was created using the “create shell” function. An offset was applied to improve the passivity of the support and to eliminate any undercuts.

A solid cylinder was created at the burs’ location using Boolean subtraction with the burs CAD previously designed and positioned in Nomad Sculpt to form the sleeves.

The resulting sleeves were connected to the tooth-supporting portion using the bar function. The intraoral scan model was subtracted from the Boolean connection between the tooth-supporting portion, the sleeve, and the bar to eliminate any interference with the mucosa and the fornices.

**FIGURE 4:**
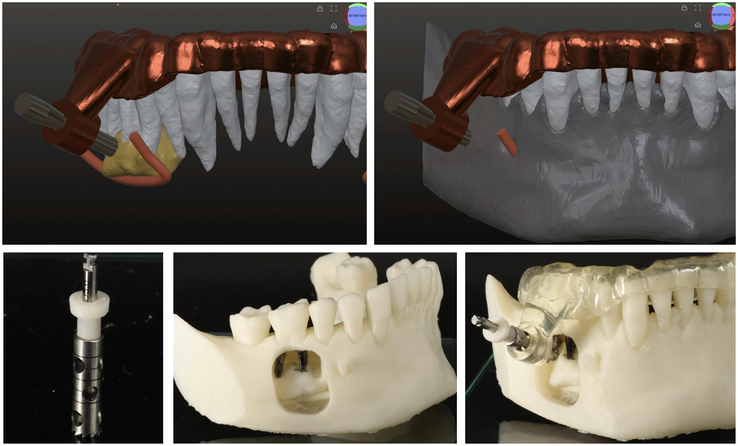
Evaluation of correct planning of the template and correct orientation of the bur using Nomad Sculpt software. A 3D-printed surgical stop drill, and a 3D stereolithography model with an inspection hatch were printed to evaluate the planning quality in the pre-clinical phase.

To ensure that the drilling stopped at the planned depth, 2 rings (different for the 2 burs) were created using Rhinoceros CAD [Robert McNeel & Associates. *Rhinoceros* (software): Robert McNeel & Associates; 2025] to prevent further advancement of the drill upon contact with the sleeve. The 2 templates thus produced were made using addition technology using a 3D stereolithography printer with a laser-based UV light source [*SprintRay Surgical Guide 3.* SprintRay Inc. (2025)] (Fig. 4).

After the administration of local anesthesia, a marginal mucoperiosteal envelope flap was performed with exposure of the posterior mandibular body. This approach was necessary to allow deeper placement of the 3D-printed surgical guide, due to the deep location of the lesion and because, during maximal mouth opening, the vestibular fold caused displacement and instability of the template.

**FIGURE 5:**
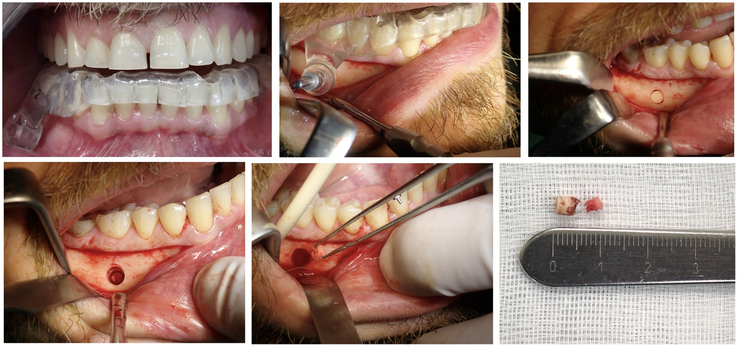
Surgical workflow of a guided biopsy

Once the guide was properly positioned on the mandibular arch, bone trepanation was carried out using a first trephine bur to the predetermined depth, aided by a mechanical stop. A second trephine was then used to core out the lesion. The bone specimen was fractured and removed, then sent to the pathology department for histopathologic examination, which confirmed the diagnosis of osteoma (Fig. 5).

**FIGURE 6:**
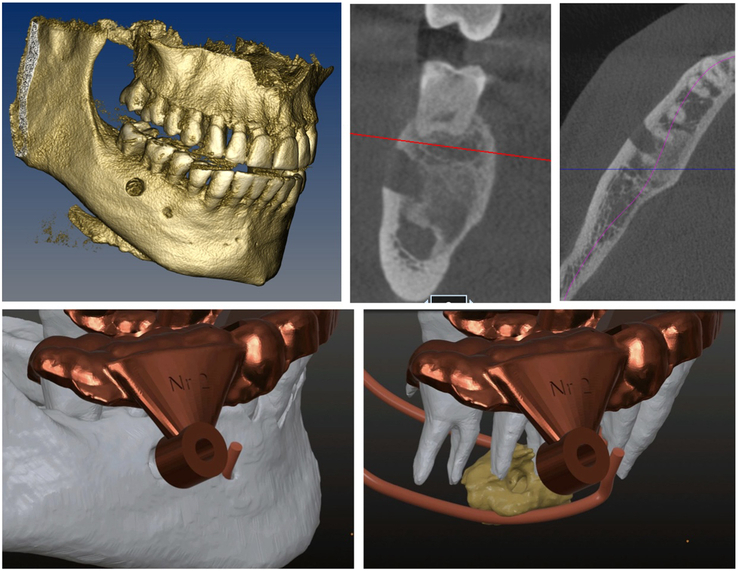
Postoperative CBCT in axial and coronal plane shows the correct removal of the pathologic tissue and the respect of the roots of tooth 4.6 and the mandibular canal. The 3D reconstruction highlights the entrance foramen. The visualization of the 3D reconstruction imported into Nomad Sculpt software demonstrates the consistency between digital planning and surgical execution.

At the end of the procedure, the surgical site was irrigated, hemostasis was achieved, and the soft tissues were closed primarily using non-resorbable silk sutures. A postoperative CBCT confirmed a safe distance between the bone window, the mandibular canal, and the roots of tooth 4.6 (Fig. 6). The postoperative course was uneventful, with no signs of hypoesthesia or paresthesia of the lower lip, nor any other reported complaints.

## DISCUSSION

The use of AI for the segmentation of the mandibular structure and the lesion, combined with digital impressions and dedicated design software, enabled the straightforward, and predictable fabrication of surgical guides. These guides assist the clinician in performing biopsies that would otherwise be highly invasive,^11^ requiring an excessive sacrifice of biological tissues both in terms of flap size and the amount of bone removed and carrying a high risk of injury to the inferior alveolar nerve or the roots of adjacent teeth involved in the lesion.

Recent studies have evaluated the accuracy of such guided biopsies, including one in vitro study^8^ and 2 clinical studies.^3,9^ All of them consistently report that surgical guides produced using desktop stereolithographic 3D printers allow for significantly higher accuracy in biopsy procedures.

The planning and design of the depth-control stops allowed for the collection of an adequate biopsy specimen, enabling the pathology department to perform a reliable histopathologic evaluation. In cases of osteosclerotic lesions, it can be challenging to harvest a sufficient amount of pathologic tissue due to the clinical similarity between bone tissue and the pathologic tissue, particularly when the lesion is located near delicate anatomic structures.

In the current surgical plan, the decision was made to use 2 coaxial trephine burs—one with an external diameter of 5 mm and the other with an external diameter of 4 mm. This choice was made for 2 main reasons: first, to increase the thickness of the osteotomy line, thus facilitating more apical positioning of the instrument to allow adequate fracture of the entire sample (given that it had different densities represented by cortical bone, medullary bone, and osteosclerotic lesion); second, because as one penetrated deeper into the lesion, the space became increasingly narrower (between the roots and MC), so only the 4 mm bur was brought to working length for lesion sampling, whereas the 5 mm bur remained 4 mm more superficial.

Postoperative CBCT imaging was used to assess the quality of the biopsy and to measure the discrepancy between the planned and the actual position of the biopsy channel. The results demonstrated good accuracy: the linear deviation of the cylinder axis was 0.6 mm, the angular deviation was 4 degrees, and the depth deviation was −0.2 mm.

Although minimal, these deviations may be attributed to cumulative errors occurring throughout the digital workflow—from segmentation and design to the fabrication of the surgical guide—as well as to slight flexion of the guide under pressure during the use of the rotary handpiece.

## References

[1] Peditto M Nucera R Rubino E. Improving oral surgery: a workflow proposal to create custom 3D templates for surgical procedures. Open Dent J 2020;14:35–44

[2] Omara M Gouda A Ali S. Computer-guided buccal cortical plate separation for removal of calcified benign odontogenic tumors affecting the mandibular angle region. Maxillofac Plast Reconstr Surg 2022;44:30 36136180 10.1186/s40902-022-00354-6 PMC9500123

[3] Lotz M Schumacher C Stadlinger B. Accuracy of guided biopsy of the jawbone in a clinical setting: a retrospective analysis. J Craniomaxillofac Surg 2021;49:556–561 33726950 10.1016/j.jcms.2021.02.025

[4] Van Hoe S Shaheen E de Faria Vasconcelos K. Contribution of three-dimensional images in the planning of cementoblastoma resection. BJR Case Rep 2021;7:20200156 34131494 10.1259/bjrcr.20200156 PMC8171136

[5] Lo Russo L Mariani P Zhurakivska K. Three-dimensional accuracy of surgical guides for static computer-aided implant surgery: a systematic review. Prosthesis 2023;5:809–825

[6] Gernandt S Tomasella O Scolozzi P. Contribution of 3D printing for the surgical management of jaws cysts and benign tumors: a systematic review of the literature. J Stomatol Oral Maxillofac Surg 2023;124:101433 36914002 10.1016/j.jormas.2023.101433

[7] Kivovics M Penzes D Moldvai J. A custom-made removable appliance for the decompression of odontogenic cysts fabricated using a digital workflow. J Dent 2022;126:104295 36116543 10.1016/j.jdent.2022.104295

[8] Postl L Mucke T Hunger S. In-house 3D-printed surgical guides for osseous lesions of the lower jaw: an experimental study. Eur J Med Res 2021;26:25 33722284 10.1186/s40001-021-00495-w PMC7958719

[9] Postl L Mucke T Hunger S. Biopsies of osseous jaw lesions using 3D-printed surgical guides: a clinical study. Eur J Med Res 2022;27:104 35780184 10.1186/s40001-022-00726-8 PMC9250179

[10] Lo HY Leung PH Su YX. AI-driven CBCT segmentation and 3D modeling of the anterior surface of maxilla for computer-assisted surgery: a comparison of multiple algorithms. J Craniomaxillofac Surg 2025;53:1683–1690 40701913 10.1016/j.jcms.2025.07.007

[11] Hu YK Xie QY Yang C. Computer-designed surgical guide template compared with free-hand operation for mesiodens extraction in premaxilla using “trapdoor” method. Medicine (Baltimore) 2017;96:e7310 28658139 10.1097/MD.0000000000007310 PMC5500061

